# Increasing Oxygen Partial Pressures Induce a Distinct Transcriptional Response in Human PBMC: A Pilot Study on the “Normobaric Oxygen Paradox”

**DOI:** 10.3390/ijms22010458

**Published:** 2021-01-05

**Authors:** Deborah Fratantonio, Fabio Virgili, Alessandro Zucchi, Kate Lambrechts, Tiziana Latronico, Pierre Lafère, Peter Germonpré, Costantino Balestra

**Affiliations:** 1 Department of Biosciences, Biotechnology and Biopharmaceutics, University of Bari Aldo Moro, 70125 Bari, Italy; deborah.fratantonio@uniba.it (D.F.); tiziana.latronico@uniba.it (T.L.); 2 Council for Agricultural Research and Economics—Food and Nutrition Research Centre (C.R.E.A.-AN), 00198 Rome, Italy; pgermonpre@gmail.com; 3 Environmental, Occupational, Ageing (Integrative) Physiology Laboratory, Haute Ecole Bruxelles-Brabant (HE2B), 1180 Brussels, Belgium; azucchi@he2b.be (A.Z.); klambrechts@he2b.be (K.L.); plafere@he2b.be (P.L.); 4 Laboratoire de Parasitologie, Faculté de Médecine, Université Libre de Bruxelles, 1050 Brussels, Belgium; 5 Department of Anaesthesiology, Erasme University Hospital, Université Libre de Bruxelles, 1070 Brussels, Belgium; 6 Hyperbaric Centre, Queen Astrid Military Hospital, 1120 Brussels, Belgium; 7 Motor Sciences Department, Physical Activity Teaching Unit, Université Libre de Bruxelles, 1050 Brussels, Belgium

**Keywords:** pulsed hyperoxia, HIF-1α, NRF2, NF-κB, hyperbaric oxygen, relative hypoxia

## Abstract

The term “normobaric oxygen paradox” (NOP), describes the response to the return to normoxia after a hyperoxic event, sensed by tissues as oxygen shortage, and resulting in up-regulation of the Hypoxia-inducible factor 1α (HIF-1α) transcription factor activity. The molecular characteristics of this response have not been yet fully characterized. Herein, we report the activation time trend of oxygen-sensitive transcription factors in human peripheral blood mononuclear cells (PBMCs) obtained from healthy subjects after one hour of exposure to mild (MH), high (HH) and very high (VHH) hyperoxia, corresponding to 30%, 100%, 140% O_2_, respectively. Our observations confirm that MH is perceived as a hypoxic stress, characterized by the activation of HIF-1α and Nuclear factor (erythroid-derived 2)-like 2 (NRF2), but not Nuclear Factor kappa-light-chain-enhancer of activated B cells (NF-κB). Conversely, HH is associated to a progressive loss of NOP response and to an increase in oxidative stress leading to NRF2 and NF-kB activation, accompanied by the synthesis of glutathione (GSH). After VHH, HIF-1α activation is totally absent and oxidative stress response, accompanied by NF-κB activation, is prevalent. Intracellular GSH and Matrix metallopeptidase 9 (MMP-9) plasma levels parallel the transcription factors activation pattern and remain elevated throughout the observation time. In conclusion, our study confirms that, in vivo, the return to normoxia after MH is sensed as a hypoxic trigger characterized by HIF-1α activation. On the contrary, HH and VHH induce a shift toward an oxidative stress response, characterized by NRF2 and NF-κB activation in the first 24 h post exposure.

## 1. Introduction

The survival in a relatively variable environment requires the ability to react to potentially stressful conditions and overcome them by triggering cellular adaptive compensatory changes and complex “system” responses [1]. Specific environmental conditions and specific events can be significantly associated to variation of oxygen availability, far from a tight “optimum”.

Accordingly, cells efficiently respond to even minute changes of oxygen availability by a specific transcriptional response targeting to counter oxygen deficiency (hypoxia), a circumstance occurring when oxygen demand exceeds its availability or, more dramatically, in the presence of a severe impairment of blood delivery, eventually resulting in ischemia. Human tissues undergo hypoxia during sepsis, sleep apnea, chronic obstructive pulmonary disease (COPD), diabetic kidney disease and wound healing, and in the context of the solid tumor microenvironment [2,3].

Conversely, in specific circumstances the organism is exposed to oxygen “overflow”. This is known to occur in association with high blood flow in skeletal muscle due to a heavy workout [4], while breathing high partial pressures of oxygen, or during hyperbaric therapy, and also during scuba diving [5,6]. These events have been reported to induce a condition frequently referred as “oxidative stress” [7] a term generally referring to “an imbalance between oxidants production and antioxidant and repair defenses, resulting in the increased steady-state levels of oxidized cellular macromolecules” [8].

According to the direction of environmental changes in oxygen availability, cells react with definite, relatively rapid and efficient cellular mechanisms to adapt, mainly relying on the rapid, pivotal effect of two transcription factors activated by either negative or positive fluctuations of oxygen availability, the Hypoxia-inducible factor 1α (HIF-1α) and Nuclear factor (erythroid-derived 2)-like 2 (NRF2), respectively.

The delicate balance between the cellular response mediated by these two transcription factors is further finely tuned by a third transcription factor, NF-κB that plays a central role in determining the result of the response to oxygen fluctuation and to cellular injury [9].

We have previously demonstrated that pulsed hyperoxia induces HIF-1α activation and the expression of genes involved in the response to low oxygen describing a “normobaric oxygen paradox” (NOP), i.e., that relative changes of oxygen availability, rather than steady-state hypoxic (or hyperoxic) conditions, coordinate HIF-1α transcriptional effects, [10,11]. This phenomenon nowadays has several different names, either “Hyperoxic-Hypoxic Paradox” [12] or “Normobaric Oxygen Paradox” [13], depending on the range of variation of PO_2_ imposed; nevertheless, a general term could be a “relative hypoxia” without reaching tissue hypoxic levels [13].

Pure O_2_ breathing has been utilized to induce a NOP effect, associated with the increase in newly synthesized erythropoietin (EPO) targeting the treatment of anemia [14,15,16]. This protocol has been further confirmed in a more recent study, conducted in a clinical setting on acutely ill patients [17].

However, clinical trials dealing with this matter are rare [18,19] and other studies have provided contradictory results, possibly due to different criteria and protocols in subjects’ selection and treatment [20,21].

At present, available data suggest that NOP effects are specifically dose and time dependent. The mechanisms underlying these effects at the molecular level are still only partially understood and this lack of knowledge limits the exploitation of this promising protocol in several different medical conditions.

Herein, we report the time trend of activation of oxygen sensitive transcription factors, HIF-**1**α, NRF2 and Nuclear Factor kappa-light-chain-enhancer of activated B cells (NF-κB), associated with NOP response in human peripheral blood mononuclear cells (PBMCs) obtained from twelve healthy subjects after one hour exposure to mild (MH), high (HH) and very high (VHH) hyperoxia, corresponding to 30%, 100%, 140% O_2_, respectively. Intracellular levels of glutathione (GSH), largely dependent on NRF2 signal [22] and the expression of the oxygen sensitive Matrix metallopeptidase 9 (MMP-9) [23], have been also assessed to better characterize the interplay between transcription factors involved in NOP response.

## 2. Results

### 2.1. Pulsed Mild Hyperoxia, but Not High Hyperoxia, Induces HIF-1α Nuclear Transfer in PBMC

**Figure 1:**
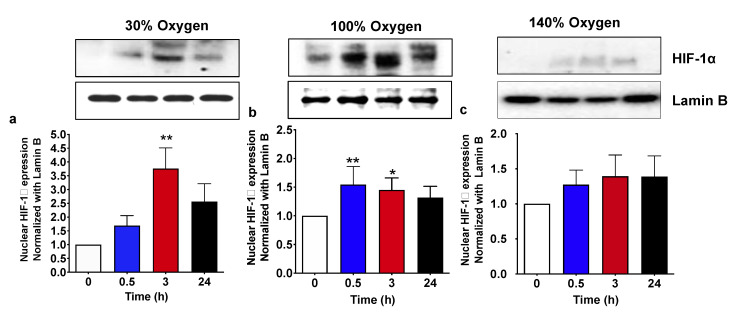
HIF-1α nuclear translocation following 1 h hyperoxia. (**a**) Mild hyperoxia (30% O_2_); (**b**) high hyperoxia (100% O_2_); (**c**) very high hyperoxia (140% O_2_) before and after the recovery to normoxic conditions. Above histograms, the picture shows a representative western blot analysis. Results are expressed as fold change (mean ± SEM) in comparison to baseline (0), which was set at 1. * *p* < 0.05, ** p < 0,01; for one-way ANOVA followed by Dunnett’s post hoc test.

We have previously demonstrated that the administration of a pulsed hyperoxia induces a “paradoxical” hypoxic response characterized by the HIF-**1**α activation both in cultured cells and in vivo, in humans [10,11,15]. The study presented herein confirms that after a period of both mild and severe hyperoxia, once returning to normal oxygen levels, all subjects experience an increase in nuclear HIF-**1**α levels. Figure 1 shows the HIF protein level in the nucleus at 30 min, 3 and 24 h after the return to normoxia. When administered at 30% oxygen, nuclear HIF-**1**α significantly increased, up to about 4-fold, with respect to the background level at 3 h, and returned back to a point close to the baseline at 24 h from hyperoxic treatment (Figure 1a). Conversely, when high hyperoxia (100% O_2_) was administered, we observed a significant early increase in HIF-**1**α nuclear transfer starting from 30 min from the return to normoxia. HIF-**1**α increase, which was of a lesser extent in comparison with the treatment with 30% oxygen, remained significantly higher than the baseline at 3 h, slowly returning close to the baseline at 24 h from hyperoxia (Figure 1b). Finally, the administration of a very high hyperoxia (140% O_2_) was associated with non-significant changes of HIF-**1**α nuclear levels at all experimental times, suggesting new actors involved in cellular response to high oxygen concentration exposure (Figure 1c).

### 2.2. Pulsed Hyperoxic Treatment, Significantly Affects NRF2 Nuclear Transfer in PBMC

**Figure 2:**
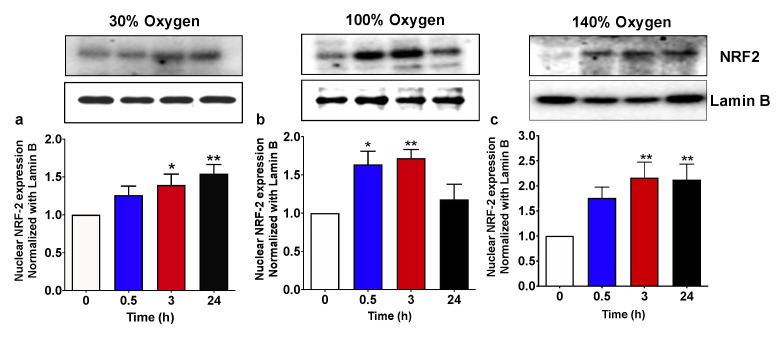
NRF2 nuclear translocation following 1 h hyperoxia. (**a**) Mild hyperoxia (30% O_2_); (**b**) high hyperoxia (100% O_2_); (**c**) very high hyperoxia (140% O_2_) before and after the recovery to normoxic conditions. Above histograms, the picture shows a representative western blot analysis. Results are expressed as fold change (mean ± SEM) in comparison to baseline (0), which was set at 1. * *p* < 0.05, ** p < 0,01; for one-way ANOVA followed by Dunnett’s post hoc test.

High oxygen breathing is known to be associated with an imbalance of redox status [24,25,26]. As mentioned in the introduction, NRF2 coordinates adaptive responses to diverse forms of stress associated with the high oxygen flux and upregulates the repair and degradation of damaged macromolecules [9,27]. Figure 2 shows that, in agreement with previous studies, breathing high oxygen was associated to a significant increase in NRF2 nuclear levels at all the tested concentrations. When administration of 100% oxygen was provided, the NRF2 level increase started at 30 min after the recovery to normoxia and returned back to the baseline at 24 h (Figure 2b). Conversely, when 30% and 140% were administered, NRF2 transfer to the nucleus occurred at 3 h from treatment and was maintained up to 24 h (Figure 2a,c).

### 2.3. High and Very High Hyperoxia but Not Mild Hyperoxia Activate NF-KB Nuclear Traslocation in Human PBMCs

Cellular response to oxygen is finely tuned by a complex interplay between a network of transcription factors able to sense and react to either minute or frankly stressful variation, setting up the system to adapt. The interaction between the HIF/NRF2 axis with the transcription factor NF-κB has already been reported in different physiological and pathological contexts [9,28,29]. In particular, following the exposure to oxidant molecular species or an increase in oxygen flux inducing a condition of oxidative stress, NF-κB may be a potential target to confer either cell protection in synergy with HIF/NRF2, or direct cellular response toward a more severe programmed cell death when the stress overwhelms the system capacity to adapt [30].

**Figure 3:**
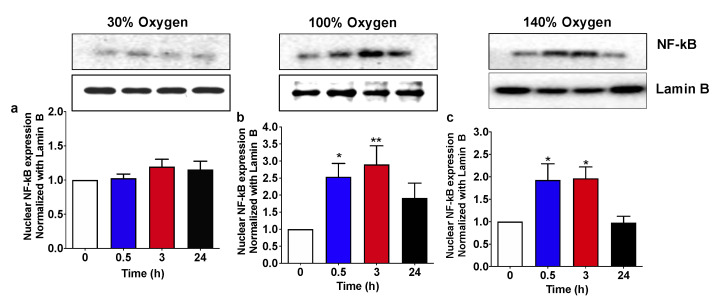
NF-κB (p65 subunit) nuclear translocation following 1 h hyperoxia. (**a**) Mild hyperoxia (30% O_2_); (**b**) high hyperoxia (100% O_2_); (**c**) very high hyperoxia (140% O_2_) before and after the recovery to normoxic conditions. Above histograms, the picture shows a representative western blot analysis. Results are expressed as fold change (mean ± SEM) in comparison to baseline (0), which was set at 1. * *p* < 0.05, ** *p* < 0,01; for one-way ANOVA followed by Dunnett’s post hoc test.

Following the administration of mild hyperoxia (30% O_2_) the nuclear levels of p65 protein subunit inside the nucleus, indicating NF-κB activation, are not significantly increased at any of the experimental time points (Figure 3a). Conversely, breathing at high (100% O_2_) and very high hyperoxia (140% O_2_), p65 nuclear levels significantly increased starting from 30 min after the recovery to normoxia and returned back to the baseline at 24 h time point (Figure 3b,c, respectively).

### 2.4. Pulsed Hyperoxic Treatment Modulates Plasma Total Glutathione Levels

Glutathione is a tripeptide that plays a pivotal role as a cofactor for a number of enzymes involved in cellular protection and recovery from different challenges, including oxidative stress. Glutathione is also involved in the formation and maintenance of disulfide bonds in proteins and in cellular death [31]. According to its role in homeostatic response, GSH synthesis is under the control of NRF2 [32] and contributes to its activation by regulating the intracellular thiols’ oxidation.

**Figure 4:**
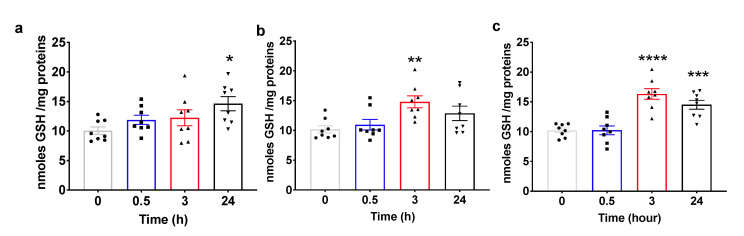
Changes in intracellular glutathione levels in human PBMC from different groups of healthy subjects after one hour of oxygen exposure in (**a**) mild (30% O_2_), (**b**) high (100% O_2_) and (**c**) very high hyperoxia (140% O_2_). The histograms display the nmole of GSH for mg of protein (mean ± SEM) in comparison to baseline (Time 0), which was set at 10. * *p* < 0.05 , ** *p* < 0.01, *** *p* < 0.001, **** *p* < 0.0001 vs. base line before oxygen exposure (time 0), for one-way ANOVA followed by Dunnett’s post hoc test.

Intracellular GSH levels follow the trend of NRF2 activation. Figure 4a shows that GSH remains at the baseline level and slightly increases only at 24 h from the recovery to normoxia in subjects treated with a mild hyperoxia (30% O_2_), paralleling the sustained increased activation of NRF2 from 3 h to 24 h from treatment. Conversely and according to a more rapid increase in NRF2 activation, intracellular GSH levels of subjects treated with high hyperoxia (100% O_2_) are significantly increased at 3 h from the recovery to normoxia and return to the baseline at 24 h from treatment (Figure 4b). Finally, when subjects are administered with a very high hyperoxia (140% O_2_), cellular GSH levels are significantly elevated at 3 and 24 h from treatment, overlapping with NRF2 activation (Figure 4c).

### 2.5. Pulsed Hyperoxic Treatment Modulates MMP-9 Release and Activity in Human Plasma

Matrix metallopeptidase 9 (MMP-9), is an enzyme that belongs to the zinc metalloproteinases family involved in the degradation of the extracellular matrix. MMPs can cleave or degrade proteins, clotting factors, chemotactic molecules, latent growth factors, cell surface receptors, and cell-cell adhesion molecules. Out of 24 MMPs, MMP9 is the only one that is undetectable in healthy tissues and highly expressed in inflammation and in several diseases, including cancer [33].

**Figure 5:**
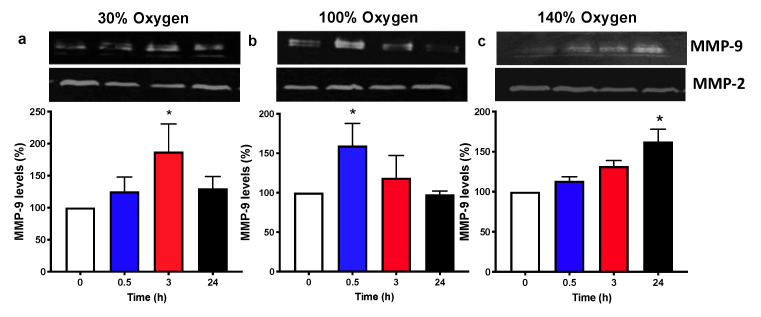
Zymographic analysis of matrix metallo-proteinase (MMPs) in human plasma from different groups of healthy subjects after one hour of oxygen exposure in (**a**) mild (30% O_2_), (**b**) high (100% O_2_) and (**c**) very high hyperoxia (140% O_2_). The pictures show a representative zymograph gel for all the subjects submitted to the analysis. The histograms display the percentage (mean ± SEM) in comparison to baseline (Time 0), which was set at 10. * *p* < 0.05 vs. base line before oxygen exposure (Time 0), for one-way ANOVA followed by Dunnett’s post hoc test.

Several studies have examined the effect of hyperbaric oxygen (HBO) on MMP-9 expression [34] and showed that its expression is dependent on the activation of the NF-kB/c-fos pathway [23], which is in turn dependent on the state of activation of the HIF/NRF2 axis [9,35]. MMP-9 plasma levels were modulated according to oxygen concentrations and time of recovery to normoxic conditions. In particular, we observed an increase in MMP-9 levels after 3h following the administration of mild hyperoxia (30% oxygen) and a recovery to basal levels thereafter (Figure 5a). The administration of high oxygen (100% oxygen) was associated with an increase in MMP-9 levels starting from 30 min and returning back to the initial levels at 3 and 24 h time points (Figure 5b). The administration of very high hyperoxia (140% oxygen) resulted in MMP-9 levels increasing in a constant trend, which became statistically significant at 24 h after recovery to normoxia (Figure 5c).

## 3. Discussion

Cellular oxygen status is a key regulator of several important biological functions. Hypoxia-inducible factor-1 (HIF-1α) is an important transcription factor regulating several genes as a response to low oxygen [9], allowing growth under hypoxia and proteins that assist hypoxic tissues to re-establish oxygen supply. We have previously reported the “normobaric oxygen paradox” as a physiological response to normoxia following a hyperoxic event. This variation is sensed by tissues as an oxygen shortage that results in up-regulation of HIF-1α activity that stimulates the increase in endogenous erythropoietin (EPO) production by creating a state of relative hypoxia (hence the term “Normobaric Oxygen Paradox”) [13]. However, the role of reactive oxygen species generated under hypoxia in triggering HIF transcriptional activity is still controversial. We have proposed a mechanism for this response based on the hypothesis of a depletion of reduced glutathione due to the increase in generation of oxygen reactive species during hyperoxia [11,36], according to previous reports indicating that that reactive oxygen species can stabilize HIF-1α [37].

It has been well established that high levels of oxygen are associated with the generation of reactive oxygen species and induce oxidative challenge to biological systems, eventually resulting in cell dysfunction/loss of function and death [38]. In the presence of these conditions, cells respond to dispose and possibly repair damages induced by the increased concentration of reactive oxygen and nitrogen molecular species inducing cytoprotective and detoxifying enzymes. NRF2 is probably one of the most important transcription factors involved in the activation of this redox-sensitive gene regulatory network.

The exact level of increased oxidative metabolism/stress needed to switch from a positive trigger to an increased oxidative stress is actually not clearly known.

Intermittent low level oxidative stresses (such as physical activity) are known to increase antioxidant protection in the presence of oxidative stress induced through bouts of acute exercise [39]. Similarly, repeated sessions of hyperbaric oxygen therapy, even inducing an oxidative stress, have been reported to be beneficial to different health parameters and associated with a significant increase in HIF [40]. Interestingly, the same reactions on telomeres can be elicited by very high or moderate exposure to high oxygen [40,41,42], suggesting that more research is needed on this topic.

In our study we did not directly address the consequences of hyperoxia in terms of “oxidative damage” or directly assess the generation of reactive oxygen (and nitrogen) species. It is, in fact, known that elevated oxygen can induce an oxidative challenge to biological systems, eventually resulting in cell dysfunction/loss of function and death due to an overwhelming generation of reactive oxygen and nitrogen species [38]. This has been reported in several physiological and pathological conditions, including high intensity physical workouts [4], and it is particularly evident in studies conducted in newborns submitted to high oxygen therapy after preterm birth [43].

However, the activation of NRF2 and NF-κB transcription factors and the paralleled increase in the level of cellular glutathione and plasmatic MMP-9 protein indirectly, but robustly, indicate the occurrence of a significant alteration of the redox status at the system level.

HIF-1α and NRF2 have been reported to interplay with a key transcription factors in inflammation and cellular response and, in particular, with NF-κB. Many studies reported that a variety of treatments suppressed the NF-κB pathway and were able to activate NRF2, clearly suggesting a crosstalk between these two transcription factors [44].

Although it is not clear yet if the suppression of NF-kB signaling and the activation of the NRF2 pathway are independent or regulated by an upstream controller, in general, with few exceptions, NF-κB and NRF2 apparently play opposite roles in cellular response. Finally, under physiological conditions, the interference of NF-κB with NRF2 transactivation may serve as a negative regulatory mechanism for the fine tuning of NRF2-ARE signaling.

The cellular response to oxygen is finely tuned by a complex interplay between a network of transcription factors able to sense and react to either minute or frankly stressful variation, setting up the system to adapt. The interaction between the HIF/NRF2 axis with the transcription factor NF-κB has already been reported in different physiological and pathological contexts [9,28,29]. In particular, following the exposure to oxidant molecular species or an increase in oxygen flux inducing a condition of oxidative stress, NF-κB activation may be a potential target to confer either cell protection in synergy with HIF/NRF2, or direct cellular response toward a more severe programmed cell death when the stress overwhelms the system capacity to adapt.

Despite this evidence, to date, the molecular mechanism regulating the oxygen response to different PO_2_ that may trigger the expression of EPO in a hypoxia-like fashion in humans is not fully understood.

In this study, we have investigated the activation of oxygen sensitive transcription factors in PBMCs obtained from humans after breathing increasing PO_2_, generating a mild, a high or a very high hyperoxia (30%, 100%, 140% O_2_, respectively). PBMC have been obtained from blood collected at 30 min, 4 h and at 24 h from hyperoxic treatment.

The return to normoxia after one hour of breathing 30% O_2_ induced a significant activation of HIF-1α starting at 3 h, returning close to the baseline at the 24 h time point. NRF2 activation followed a similar trend, being significantly activated at 3 h from the recovery to normoxia and remaining slightly, but significantly activated up to the end of the observational period. Conversely, NF-κB was not affected at any of the experimental times.

GSH intracellular levels and plasma MMP-9 mirrored the activation of NRF2 and HIF-1α, being significantly increased at 24 h and at 3 h, respectively. These activation profiles suggest that 30% oxygen induced a “clean” NOP characterized by a mild but sustained antioxidant response and a sharp, temporary activation of HIF-1α in the absence of a NF-κB response [45].

The administration of 100% oxygen for one hour was associated with a rapid HIF-1α response starting at 30 min from the recovery to normal oxygen concentration, with a progressive return to pre-treatment levels at 24 h. Differently to the administration of a MH, HH induced a significant activation of NF-κB starting at 30 min from hyperoxic treatment, which was not detectable at 24 h. Additionally, in this case, both GSH intracellular levels and MMP-9 in plasma overlapped the activation profile of NRF2 and HIF-1α, respectively. In this case, the overall response can be described as a combination of a NOP with oxidative stress response, characterized by the simultaneous activation of both NRF2 and NF-κB, an early activation of HIF-1α rapidly returning to the baseline level and the synthesis of GSH, as an antioxidant response, and MMP-9 aiming to cope with cellular stress response [46].

Finally, the administration of a hyperbaric hyperoxia corresponding to 140% of oxygen, with respect to “normal” conditions, was associated with an evident oxidative stress component. After hyperbaric oxygen, NOP is not any longer evident and HIF-1α remains at the baseline levels. Conversely, both NRF2 and NF-κB are significantly activated starting at 3 h and 30 min, respectively. NF-kB returns to baseline at the 24 h time point while NRF2 remains activated up to the end of the experimental window. After hyperbaric oxygen, both cellular GSH and plasma MMP-9 levels significantly increase, indicating that the treatment induced a significant oxidative stress at the system level.

In addition, at the systemic level, HIF activation associated with both 30% and 100% pO_2_ was accompanied by an increase in EPO levels of about 10% at 24 h from oxygen administration, with respect to the pre-treatment (data not shown). This increase overlaps with the increase in circulating EPO following a pulsed hyperoxia already reported by our group. In fact, we have already demonstrated that EPO increases after 100%, 50% [47] and 40% [48] at 24 h from high oxygen breathing. These original data were the conceptual basis for the conceptualization of the “normobaric oxygen paradox” [10].

Interestingly, and further confirming the “paradoxical” response to the return to normoxia after high oxygen, we also observed that chronic exposure to high (100%) oxygen breathing has a similar effect on hemoglobin levels to that of hypoxia (15% oxygen) [48,49].

In general, in our study, we hypothesized that the response mediated by oxygen-sensitive transcription factors was fundamentally determined by the oxygen partial pressure independently of the strategy (normobaric vs. hyperbaric) of administration. In fact, it has been shown [50] that the oxygen partial pressure, but not inert gas pressure or total pressure, is the main determinant of ROS production. Although we cannot formally exclude possible interactions between inert gas pressures and reactions further down the transcription factor chain of events, this appears to not be relevant to our experimental setup where subjects submitted to “very high hyperoxia” (corresponding to 140% pO_2_) breathed pure oxygen at 1.4 ATA, with no inert gas. Our observations strongly indicate the presence of a *continuum* of effects paralleling partial pressures of oxygen.

However, the hypothesis that pressure, independently of pO_2_, would exert an effect on the activation of oxygen sensitive transcription factors is surely of great anticipation and needs specifically designed experimental protocols. Nonetheless, it is considered to be outside of the scope of our study and of this report.

## 4. Conclusions

In conclusion, our study confirms that, in vivo, the return to normoxia after MH is sensed as hypoxic stress characterized by HIF-1α activation. On the contrary, HH and VHH induce a shift toward an oxidative stress response, characterized by NRF2 and NF-κB activation in the first 24 h post exposure.

Even though it is possible that higher levels of hyperoxia (HH, VHH) may induce late responses to recover homeostasis over a longer window of time, previous studies in cultured cells [11] and in vivo [15,16,17] suggest that important adaptive responses occur within shorter times. However, further investigations are needed to investigate if pulsed hyperoxia induces specific compensatory reactive adaptations at longer periods. Future studies should focus on the two components of this paradigm: oxygen exposure (time and PO_2_) and time between sessions (intermittent exposures) [15,16,18].

Overall, our data suggest the occurrence of an “hormetic” adaption to high oxygen triggered by the activation of signaling cascades leading to the expression of antioxidant systems and are in agreement with data obtained in rodents undergoing either hyperbaric or normobaric oxygen, initially inducing a significant oxidative stress, which was eventually resolved after a set of continued exposures [51].

In order to optimize useful clinical protocols from this paradigm, future studies are expected to focus also on “down-stream” effects of HIF-1α transcriptional activation. Depending on the therapeutic target, using MH may be more desirable [16,30] or, on the other hand, eliciting an oxidative stress by means of HH/VHH administration may be considered a more desirable effect [40].

## 5. Materials and Methods

### 5.1. Experimental Protocol

Twelve healthy subjects (4 females and 8 males), physiotherapy students aged 21.8 ± 2.3 and 21.25 ± 2.1 years old (mean ± SD), with 1.75 m ± 6.6 height and 69.0 ± 8.7 kg weight, participated in this study after Ethics Committee approval from the Bio-Ethical Committee for Research and Higher Education, Brussels (N° B200-2_02_0-088) and written informed consent was obtained. Participants were prospectively randomized into three groups, each comprising 4 persons, and exposed to different oxygen PO_2_ for 1 h.

The first group received 30% O_2_ (0.3 bar; 300 hPa PO_2_) by means of an orofacial non-rebreather mask with a reservoir; the breathing gas flow (from a pressurized gas tank) was set at 10 L/min, with care being taken to fit and tighten the mask on the subject’s face. Group two received 100% O_2_ (1.0 bar, 1000 hPa PO_2_) from an oxygen concentrator (NewLife Intensity, CAIRE Inc., Ball Ground, GA, USA) with a similar non-rebreathing mask setup. Group three received 140% O_2_ (1.4 bar, 1400 hPa PO_2_), which was placed in a one-person hyperbaric chamber (Biobarica, Buenos Aires, Argentina).

Venous blood samples were collected at baseline (before oxygen exposure) 30 min, 3 h and 24 h after exposure, then later. Subjects were instructed not to take any medication or perform strenuous physical exercise 24 h before or stay in altitude up to 2 weeks before and during the entire study protocol and until blood collection was complete. Fifteen milliliters of blood were collected in Electrochemiluminescence (EDTA) tubes for separation of plasma and peripheral blood mononuclear cells (PBMCs) before oxygen breathing (time 0) as well as at 0.5, 3 and 24 h after exposure to hyperoxia. Absence of hemolysis in plasma was confirmed by measuring the absorbance of plasma at 414 nm, using an absorbance of 0.2 as cut-off.

### 5.2. Nuclear Lysate Preparation and Western Blotting Analysis

Nuclear lysate from human PBMC was prepared as previously described by [52]. Twenty μg of nuclear proteins, quantified with the Bradford method (Bio-Rad Laboratories Inc., Hercules, CA, USA), were separated by gel electrophoresis on 4–12% Bis-Tris Criterion XT precast gels (Bio-Rad Laboratories Inc., Hercules, CA, USA) and electroblotted onto polyvinylidene fluoride membranes (Amersham Pharmacia Biotech Inc., Piscataway, NJ, USA). Immunoblotting was performed with rabbit HIF-1α antibody (1:1000), anti-NRF2 polyclonal antibody raised against a peptide mapping at the C-terminus (C-20) (1:1000), rabbit anti-NF-κB (p65) polyclonal antibody (1:1000) (Santa Cruz Biotechnology, Dallas, TX, USA), mouse anti-Lamin B monoclonal antibody (Santa Cruz Biotechnology, Dallas, TX, USA) (1:1000), followed by peroxidase-conjugated secondary antibody HRP labeled goat anti-rabbit Ig (BD Pharmigen, San Diego, CA, USA) (1:5000), goat anti-mouse IgM secondary antibody HRP conjugate (Thermo Scientific, Waltham, MA, USA) (1:10,000), and visualized with an Electrochemiluminescence (ECL)western blotting system (AmershamBiosciences, Buckinghamshire, UK).

### 5.3. Intracellular-Reduced Glutathione (GSH) Assay

Intracellular reduced glutathione (GSH) was determined using Ellman’s reagent (5-5′-dithiobis 2-nitrobenzoic acid, DTNB) as previously described [44]. Briefly, cell lysates were deproteinized with 10% sulfosalicylic acid and centrifuged at 17,000× *g* for 5 min at 4 °C. The supernatant was collected and then diluted with 0.2 M sodium phosphate buffer (pH = 8.0). Later, an equal volume of 0.6 mM DTNB was added and, after 10 min, the optical density of the yellow-colored complex was measured. A standard curve was obtained with pure GSH. The intracellular levels of GSH were normalized with the protein content determined with the Bradford assay and expressed as nmol/mg protein. Each analysis was carried out in triplicate.

### 5.4. Zymographic Analysis of Matrix Metalloproteinase (MMP)-2 and -9 Plasma Levels

Plasma levels of MMP-2 and MMP-9 were detected by zymography as reported by [53]. Briefly, 3 µl of plasma were solubilized with 10 µL of non-reducing sodium dodecyl sulfate (SDS)-sample buffer and loaded on a 7.5% polyacrylamide gel copolymerized with 0.1% (*w*/*v*) gelatin. After the electrophoretic run, gels were rinsed twice with 2.5% Triton X-100/10 mM CaCl_2_ in 50 mM Tris–HCl, pH = 7.4 and incubated for 20 h at 37 °C in developing buffer (1% Triton X-100/50 mM Tris–HCl/10 mM CaCl2, pH = 7.4) in order to reactivate the enzymes. Then, gels were stained with Coomassie brilliant blue R-250 and destained in methanol/acetic acid/H2O (4:1:5 *v*/*v*/*v*). Gelatinolytic activity of MMP-2 and MMP-9 was visualized as clear bands of digestion on the blue background of the gel and was identified by co-migration with standard MMP-2 and MMP-9 (Alexis Biochemicals, San Diego, CA, USA). Computerized densitometric image analysis, using Image LabTM Software (Bio-Rad Laboratories), was carried out to quantify MMP-2 and MMP-9 gelatinolytic activity which was expressed as optical density (OD)×mm^2^, representing the scanning area under the curves which take into account both brightness and width of the substrate lysis zone. No variation was observed for MMP-2, which is constitutively expressed in body fluids and was used as an internal control of sample processing. In this respect, to compare results taken from different gels and quantify MMP-9, the ratio of MMP-9 to MMP-2 (MMP-9/MMP-2) was calculated for each analyzed sample.

### 5.5. Statistical Analysis

Normality of data was performed by means of Shapiro–Wilk or D’agostino–Pearson tests. When a Gaussian distribution was assumed, they were analyzed with a one-way ANOVA for repeated measures with Dunnett’s post-hoc test. If the Gaussian distribution was not assumed, the analysis was performed by means of a non-parametric multiple comparisons Dunn’s test. Taking the baseline measures as 100%, percentage or Fold changes were calculated for each oxygen protocol, allowing an appreciation of the magnitude of change rather than the absolute values. All statistical tests were performed using a standard computer statistical package, GraphPad Prism version 5.00 for Windows (GraphPad Software, San Diego, CA, USA). A threshold of *p* < 0.05 was considered statistically significant. All data are presented as mean ± standard error of the mean (SEM).

## Data Availability

Data are available at request from the authors.

## Abbreviations

ANOVA	Analysis of variance
EPO	Erythropoietin
GSH	Intracellular reduced glutathione
HIF-1α	Hypoxia-inducible factor 1α subunit
MPPs	Matrix metallopeptidases
NF-κB	Nuclear Factor kappa-light-chain-enhancer of activated B cells
NOP	Normobaric Oxygen Paradox
NRF2	Nuclear factor (erythroid-derived 2)-like 2
PBMC	Peripheral Blood Mononuclear Cells
PO_2_	Oxygen Partial Pressure
